# Comparative Evaluation of Chemical Garden Growth Techniques

**DOI:** 10.1021/acs.langmuir.3c01681

**Published:** 2023-09-15

**Authors:** Bahar Aslanbay Guler, Zeliha Demirel, Esra Imamoglu

**Affiliations:** Department of Bioengineering, Faculty of Engineering, Ege University, 35100 Izmir, Turkey

## Abstract

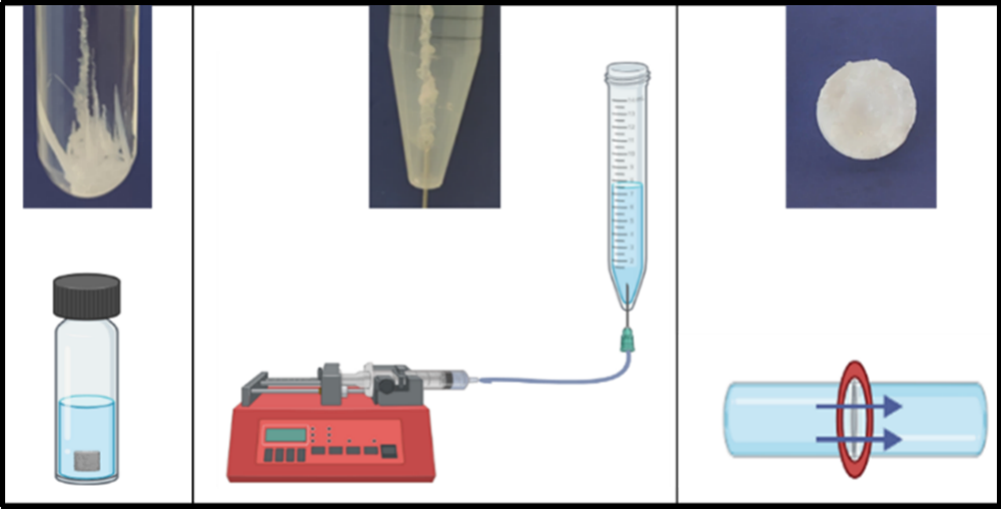

Chemical gardens are an exciting area of self-organized precipitation structures that form nano- and micro-sized structures in different shapes. This field has attracted great interest from researchers due to the specific characteristics and potential applications of these structures. Today, research on chemical gardens has provided deeper information regarding the formation mechanisms of these structures, and several techniques have been developed for chemical garden growth. However, they all show different growth patterns and lead to the formation of structures with a variety of morphological, chemical, or physical properties. This study aimed to evaluate the effects of different production techniques on chemical garden growth, taking into consideration the growth patterns, morphology, microstructure, and chemical composition. The chemical garden structures obtained in seed and injection experiments, two common methods, showed highly similar surface structures, void formation, and chemical composition. The membrane growth method has a small number of applications; thus, it was comprehensively evaluated to add new insights to the existing limited data. It produced the most stable and standard structures in a flat sheet-like shape and showed different morphologies than those observed in other two methods. Overall, this study presented significant results about the effect of growth techniques on chemical garden structures and similar systems.

## Introduction

Chemical gardens are a classical example of self-organized precipitation structures. Early reports on these systems date back to the 17th century, when Johann Glauber discovered the basic chemistry of these systems.^1^ The most iconic examples of chemical gardens in nature are hydrothermal vent chimneys. They grow as a result of precipitation processes at the interfaces between seawater and the hydrothermal fluid flowing through the vents. The leading theory behind the emergence of life on Earth raises a question: can these mineral-rich chimneys found in oceans be the first evidence of living organisms? The ongoing studies around this question have attracted the attention of scientists to the lab-grown chemical gardens, and significant progress has been made to reveal the full potential of these systems in the fields of chemistry, physics, fluid dynamics, biology, and geology.^2−4^ As part of the increasing research activities, many potential application areas such as electrochemistry, material science, microfluidics, sensors, filtration, and catalysis are suggested for the use of chemical garden structures.^5^

Today, research on chemical gardens has provided deeper information regarding the formation mechanisms of these self-organized structures. In the simplest case, a metal salt crystal is dropped into a solution containing reactive anions, commonly silicate, carbonate, phosphate, etc. The dissolving metal ions react with their anionic counterparts and form a colloidal semipermeable membrane around the salt crystal. Differences in pH and ion concentration between the two sides of the membrane cause osmotic pressure that drives the water to flow from the anionic solution toward the inside of the membrane. In the meantime, the salt continues to dissolve, leading to an increase in internal volume up to a point. Eventually, the membrane ruptures and jet fluid exits through the hole, causing the formation of new precipitates. These repeated ruptures and precipitations produce a hollow, tubular structure until the seed crystal has completely dissolved and the osmotic pump action has stopped. This process is called seed growth which is the most common and basic procedure to form chemical gardens.^6−8^ Alternatively, the injection method that includes the injection of a solution containing one reactive ion into a reservoir containing another reactive ion allows the production of tubular chemical gardens. This technique offers a more controlled growth pattern by adjusting the suitable experiment conditions for injection rate, ion concentrations, pH, and growth direction.^9−11^ Another growth method in a quite different setup is membrane growth. It consists of different types of inert matrix (dialysis tubing, parchment paper, and ion-exchange membrane) that are placed between two reactive solutions. The use of matrix allows the permeation of ions from one side of the membrane to the other, resulting in the formation of precipitation structures on the membrane.^12,13^ All of the techniques mentioned provide great information about the characteristics of chemical gardens. However, they all show different growth patterns and lead to the formation of structures with a variety of morphological, chemical, or physical properties.

The aim of this study is the comparative evaluation of chemical garden formation using three different production techniques: seed growth, injection growth, and membrane growth. The seed and injection methods are two common techniques for chemical garden production. However, membrane growth has a highly limited number of applications, which highlights the novelty of this article. Magnesium salt and silicate anion were chosen as constituents due to their importance in biomineralization and abundance on Earth.^4,14^ The morphologies, microstructures, chemical compositions, and thermal properties of the obtained structures were analyzed using scanning electron microscopy (SEM), energy-dispersive spectroscopy (EDS), X-ray micro-tomography (μ-CT), X-ray photoelectron spectroscopy (XPS), and Raman spectroscopy. This comparative evaluation of three techniques for chemical garden growth using comprehensive analyzes has been brought into the literature studies for the first time.

## Materials and Methods

### Materials

Sodium silicate (Na_2_SiO_3_) and magnesium chloride (MgCl_2_) were purchased from Sigma-Aldrich and Alfa Aesar, respectively. Solutions were prepared by dissolving the chemicals in distilled water (pH = 6.8–7, conductivity 11 μS/cm). Cellulose dialysis membrane (molecular weight cut-off 14 kDa, Sigma Aldrich) was chosen as an inert matrix for the membrane experiments. A programmable syringe pump (New Era-USA) was used for the injection experiments.

### Seed Experiment

**Figure 1:**
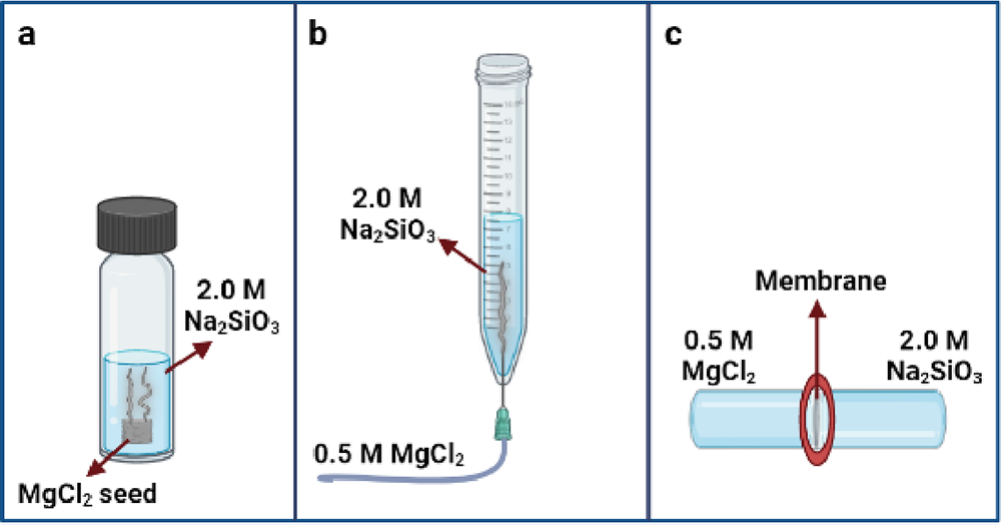
Experimental methods for chemical garden growth, (a) seed growth, (b) injection growth, and (c) membrane growth.

To prepare the salt crystal, 200 mg of MgCl_2_ was homogenized with an agate mortar and pressed into cylindrical pellets of 10 mm diameter and 3 mm thickness. The pellet was put into a cylindrical transparent vessel of 6 × 3 cm of height and diameter (Figure 1a), and 20 mL of the aqueous solution of 2.0 M Na_2_SiO_3_ (pH = 13.0) was poured carefully into the vessel, avoiding the formation of air bubbles. The crystal was left in solution for 24 h to complete the precipitation reaction. After this period, the silicate solution was removed from the vessel, obtained precipitation structures were harvested carefully, and they were dried at room temperature after washing with distilled water.

### Injection Experiment

A 0.5 M MgCl_2_ solution (4 mL) was fed into a 2.0 M Na_2_SiO_3_ solution (10 mL) by a syringe pump at a constant flow rate of 2.0 mL/h (Figure 1b). The injection was carried out for 2 h until the precipitate reached the air–liquid interface and settled down to the bottom of the vessel. After the injection was stopped, the silicate solution was removed, and the precipitate structure was harvested carefully, washed with distilled water, and dried at room temperature.

### Membrane Experiment

The setup for membrane growth experiments consisted of two adjacent vessels placed horizontally (Figure 1c). The cellulose dialysis membrane was tightly fixed between two fluid reservoirs. Each vessel was filled with 10 mL of 0.5 M MgCl_2_ solution (left side) and 10 mL of 2.0 M Na_2_SiO_3_ solution (right side) and allowed to interface across the membrane. The precipitation process was carried out for 24 h without any external interference. At the end of the period, both solutions were removed from the vessels, and the precipitate structure was separated carefully from the dialysis membrane. It was washed with distilled water and dried at room temperature.

### Characterization Analysis

The morphologies, microstructures, chemical compositions, and thermal properties of the obtained structures were analyzed using SEM, EDS, μ-CT, XPS, and Raman spectroscopy.

SEM images and EDS spectra of structures from different experiments have been analyzed using a Thermo Scientific Apreo S instrument, which was coupled with EDS (ThermoFisher Scientific, USA) at 5–25 kV. The XPS spectra were obtained with a Thermo Scientific K-Alpha (USA) spectrometer using a monochromatic Al-Kα source. The detailed survey scan analysis was carried out in the constant pass energy mode at 50 eV with a 400 μm spot size. For Raman spectroscopy, an inVia Raman system (Reinshaw Plc, UK) was used. The 532 nm excitation laser beam was focused on the samples, and spectra were collected at different positions over the range of 200–1200 cm^–1^ using a 2400 lines/mm diffraction grating. CT scans were performed using a Scanco Medical μCT50 (Switzerland) instrument. The operation parameters were as follows: 70 kV source voltage, 114 μA current, 300 ms integration time, and 5 μm pixel size. The acquired slice data was reconstructed and analyzed by using the evolution program (Scanco Medical, Switzerland) to render 3D images.

## Results and Discussion

### Growth Pattern of the Chemical Garden Techniques

**Figure 2:**
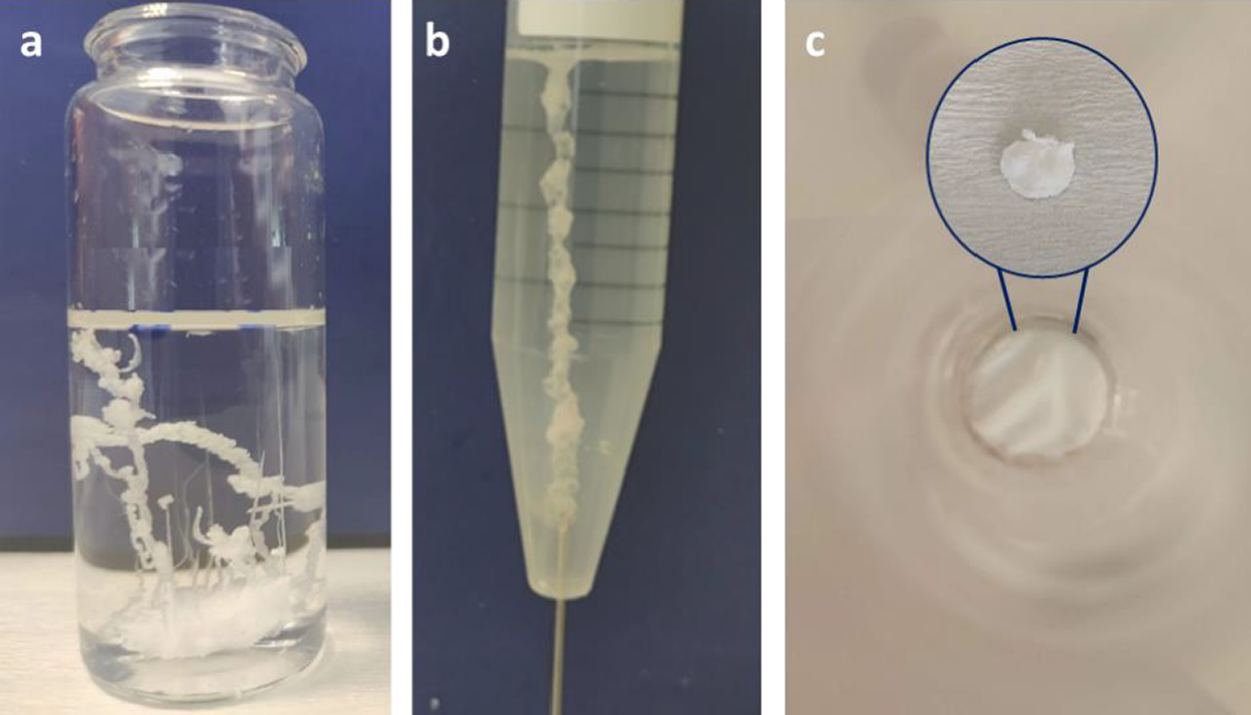
Chemical garden structures formed by (a) seed experiment (scale bar = 6.0 mm), (b) injection experiment (scale bar = 5.5 mm), and (c) membrane experiment (scale bar = 5.0 mm).

The macroscopic observations made during the experiments showed that each method resulted in different growth patterns of precipitation structures, as expected. In the seed method, an initial semipermeable membrane was formed after the alkaline solution was poured onto the MgCl_2_ crystal. After a few minutes, many irregular white tubes were observed with varying diameters between 100 and 800 μm. Tubular elongation and new precipitate formation continued rapidly until the tubes reached the air–liquid interface. Then, the overall growth rate slowed down, and the existing tubes became thicker and more intense. The final result at the end of the 24 h was a combination of numerous irregular tubular structures of different sizes, resembling the branches of a tree (Figure 2a).

When the aqueous solution of 0.5 M MgCl_2_ was fed into the 2.0 M Na_2_SiO_3_ in the injection experiments, a small white precipitate first formed at the injection point. As the flow continued, the precipitate showed vertical growth, and the morphology of the generated tube mainly consisted of bulbs, as previously reported by Barge et al.^10^ The initial hollow tube grew continuously toward the top of the tube, while some small branches were observed at random points of the main structure (Figure 2b). The precipitates reaching the fluid surface formed large clusters of magnesium silicate structure that later sank to the bottom of the vessel because of their higher density. Thus, the injection was ended after 2 h to prevent the irregular precipitate accumulation. The resultant structure was a rigid wide tube of approximately 5 cm length and 0.5 cm width.

In the membrane experiment, cellulose dialysis membrane was used as a template to provide ion transport between salt and alkaline solutions. When these two solutions were placed in two vessels connected to each other with the dialysis membrane, a white precipitate formation was observed as a flat film on the surface of the template (Figure 2c). This growth pattern was a result of the semipermeable characteristics of the dialysis membrane, which allow reactive ions to pass across the membrane but prevent the mixing of two fluids.^15^ It should be noted that the precipitate was only formed on the side toward the magnesium chloride solution, indicating that silicate ions passed across the membrane through the salt solution, and the precipitation reaction produced a magnesium silicate precipitate at this surface. After the solutions were removed, the precipitate structure attached to the dialysis membrane was collected from the system and then easily separated from the template. Based on visual observations, the chemical gardens obtained in membrane experiments showed a distinct pattern with two-dimensional precipitates^16,17^ instead of having the typical three-dimensional morphology observed in seed and injection experiments. The differences in the morphology of the chemical gardens may be attributed to the specific conditions and constraints imposed by the membrane setup, leading to variations in the growth patterns and crystal formation of the precipitates.

### Microstructural Analysis by SEM Coupled with EDS

**Figure 3:**
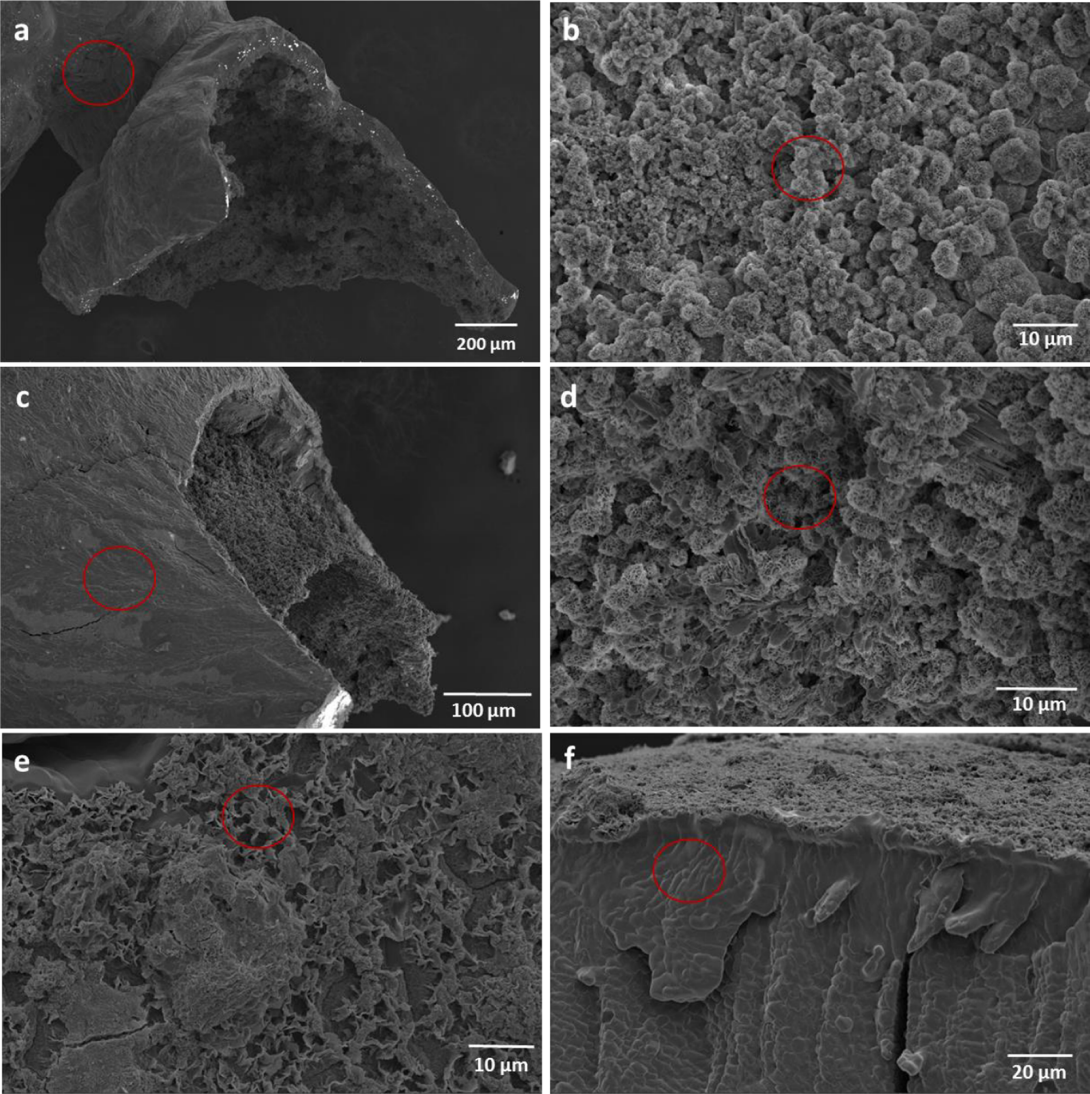
SEM images of structures obtained in the seed experiment (a, external surface; b, internal surface), injection experiment (c, external surface; d, internal surface), and membrane experiment (e, magnesium salt solution side; f, cellulose acetate membrane side). The scale bars correspond to (a) 200 μm, (b) 10 μm, (c) 100 μm, (d) 10 μm, (e) 10 μm, and (f) 20 μm. Red circles present areas interested in EDS analysis.

The microstructural properties of the obtained structures were analyzed using SEM, as shown in Figure 3. The morphologies of the samples from the seed and injection experiments shared significant similarities, which were consistent with the typical characteristics of chemical gardens.^6^ Both of them had a smooth outer surface and a complex internal surface with many heterogeneous crystals (Figure 3a,c). These crystals were uniform flowerlike or honeycomb-like particles, which were formed by a large number of thin petals connecting with each other. The petals led to the formation of a porous structure that caused an increase in the surface area of the internal part of the samples. The observed crystals on the internal surface predominantly indicated the presence of magnesium silicate and NaCl salt, which was consistent with the results from EDS analysis (Figure S1b,d) and previous reports.^4,18,19^ In contrast, the primary component found on the external smooth surface of solids formed in seed and injection experiments was magnesium oxide, while the relative quantities of magnesium silicate and sodium silicate were lower on these surfaces compared to those observed on the internal surfaces (Figure S1a,c). In the membrane experiment, it was observed that the surface morphologies were clearly different for the two sides of the precipitate: rough on the solution side (Figure 3e) and smooth on the side adjacent to the cellulose membrane (Figure 3f). The rough surface exhibited a heterogeneous topography with different-sized crystals and petal-like hierarchical structure, which corresponded to magnesium hydroxide and magnesium silicate formation. The EDS spectrum demonstrated that this surface mainly consisted of Mg, Si, and O, (Figure S1e). Also, minor quantities of Na and Cl were detected, indicating the existence of unreacted magnesium salt and the formation of NaCl. Conversely, the other side of the structure had a smooth surface that became rougher in a small portion of the area due to crystal accumulations. The chemical composition was also different from the rough surface, being mainly Na and Cl (Figure S1f). This result indicated that the ion transport through the dialysis membrane generated NaCl salt, which created a smooth layer by the orientation of NaCl crystals. Furthermore, Mg was also identified as a dominant compound on this surface, which implied the formation of a small amount of magnesium silicate and the presence of an unreacted MgCl_2_ salt.

### High Scan X-ray μCT Analysis

**Figure 4:**
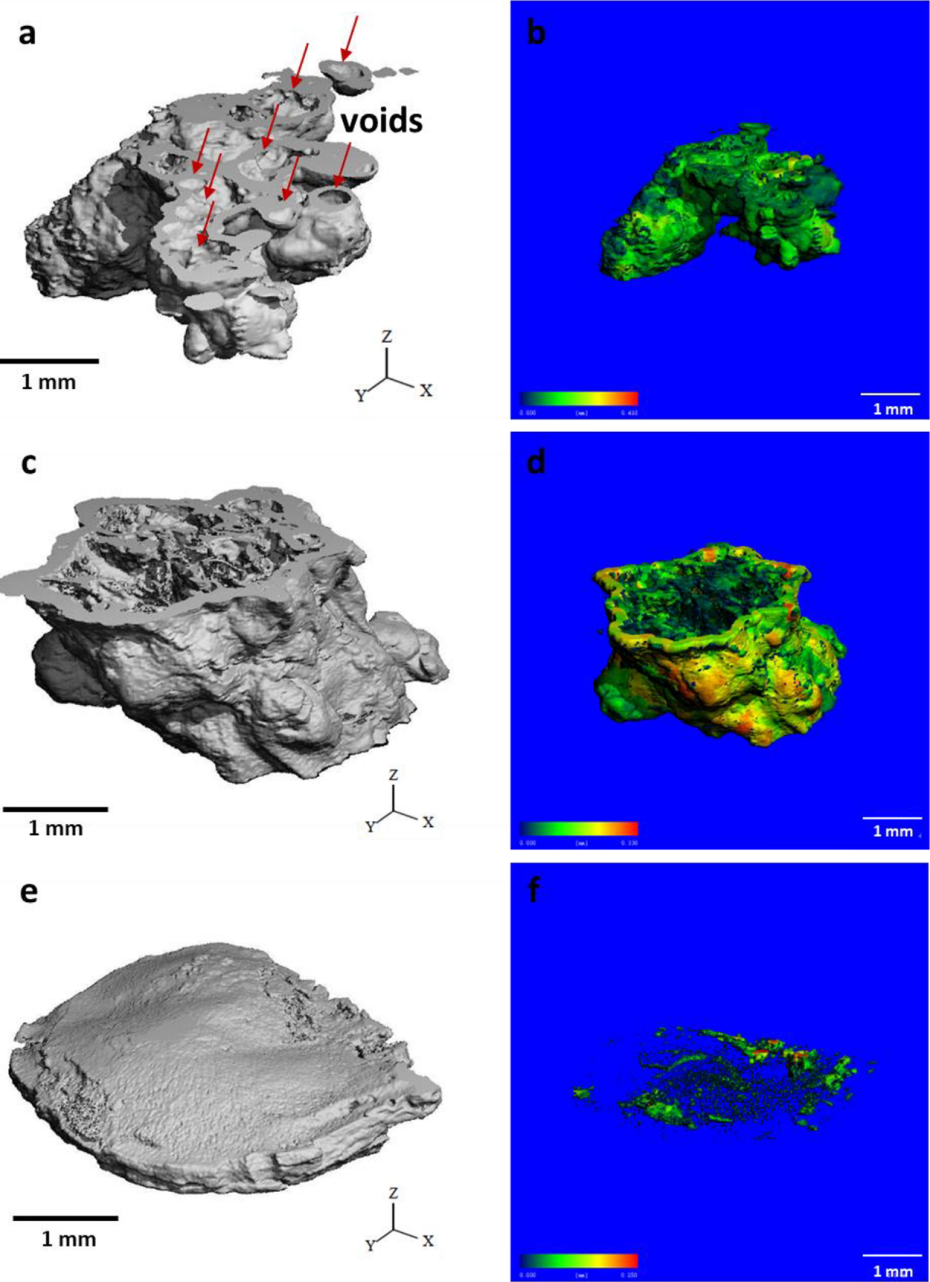
Reconstructed 3D μCT images of structures from seed (a), injection (c), and membrane (e) experiments. Wall thickness distribution of structures from seed (b) and injection (d) experiments. Void distribution of structures from the membrane experiment (f).

High-resolution μCT scanning was performed to evaluate the specific characteristics of the obtained structures, such as wall thickness, void formation, and volume fraction, by creating their three-dimensional (3D) models. For the analysis of the structures from seed and injection experiments, a small portion of samples with a maximum length of 1.5 mm was prepared to reduce processing time and provide more accurate results, whereas the sample from membrane growth was directly used since it had a simple 2D geometry. As shown in Figure 4a, the reconstructed 3D image of the structure obtained from the seed crystal exhibited a large quantity of randomly distributed and irregularly shaped voids, whose main sizes were noticed to be around 300–500 μm. The volume fraction of these voids in the region of interest, which was calculated considering solid volume and total volume (solid plus voids), was found to be 0.46. In addition, the wall thickness of this structure had a uniform distribution with an average value of 140 μm (Figure 4b). The 3D image of the structure produced in the injection experiment appeared in an irregular tubular shape that possessed a large void volume at the center of the sample (Figure 4c). As shown in the cross-section view, there were multiple thin precipitate walls inside the void that could be formed by the precipitation reaction of the interior fluid flow during the experiment. The void volume fraction was found to be 0.59, which was higher than the structure obtained from the seed experiment due to the presence of large voids. Also, the mean wall thickness value of this structure was 170 μm, which was greater than in seed growth. These differences between seed and injection growth were due to the variations in the growth mechanisms: osmosis is the primary driving force in seed growth, whereas fluid pressure derived from injection becomes more prominent in injection growth.^6^ As expected, the structure from membrane growth had a significantly different morphology (Figure 4e). It had a flat sheet-like circular shape with a mean wall thickness value of 360 μm. The void distribution, which was colored according to their sizes, was highly heterogeneous and large voids were mainly located at the edges of the structure, while small ones were found in the middle section (Figure 4f). Overall, the void volume fraction was found to be 0.23, which was 2–2.5-fold lower than obtained in the other two methods.

### Chemical Analysis of Structures with XPS and Raman Spectroscopy

XPS is a powerful tool to determine the chemical composition of a material surface using X-rays under ultra-high vacuum conditions. The wide-range survey spectra and high-resolution XPS spectra of magnesium and silica elements are presented in Figures S2 and 5, respectively. The main components in all of the three structures were Mg, Si, Na, Cl, O, and C. In more detail, the Mg 1s spectra of all samples revealed a single chemical state of magnesium at 1303.1, 1303.5, and 1303.0 eV for seed, injection, and membrane experiments, respectively. These peaks indicated the presence of Mg–O bond.^20,21^ The shift in the binding energy of Mg 1s was attributed to the hydroxylation of surface and formation of Mg(OH)_2_ component.^22,23^ When comparing the intensities of the magnesium (Mg) peaks, it became evident that the magnesium content of the structure obtained from the membrane experiment was higher compared to the other two solids formed in the seed and injection experiments. This outcome was anticipated, as the chemical garden developed in the membrane growth was formed on the side adjacent to the magnesium chloride solution, indicating a naturally elevated Mg content. According to XPS data of structure from the seed experiment, the Si 2p spectrum had one peak centered at 102.53 eV, which was identified as SiO_2_.^24^ In the Si 2p spectrum of the structure obtained in the injection growth, there were two peaks located at 103.66 and 104.89 eV. These peaks were attributed to silicon atoms in the O–Si–O and Si–OH chemical bonds, respectively.^25,26^ Moreover, the last structure from the membrane experiment showed two peaks found at 102.52 and 103.98 eV, which suggested the presence of SiO_2_.^24,27^ Although the chemical composition and bond formations were similar, multiple shifts in binding energies were observed. This suggests that the oxidation and hydroxylation of the surface influenced the chemical state of Si. Considering the intensity of Si peaks, Si–O bonds had higher values in the structure from the injection experiment, while the seed experiment led to the formation of solids with the lowest Si–O content. The higher Si–O content observed in the injection growth can be attributed to the dynamic nature of the injection method. In this method, the salt solution was continuously fed into the silicate solution for a duration of 2 h, leading to the participation of a greater number of elements in the composition of the chemical garden. For the peaks of both Mg and Si elements, the seed growth resulted in the lowest intensities due to consistent production conditions maintained during the seed growth process. Moreover, the surface composition of structures was further confirmed by O 1s XPS spectra (Figure S3). All of the structures from three experiments had three chemical states of oxygen at ∼531.4, ∼532.5, and ∼536.7 eV, which indicated the presence of O–H/Si–O, C–O, and chemisorbed oxygen/water, respectively.^28−31^ The XPS results verified that growth methods did not exhibit a significant difference in terms of chemical composition and bond formation.

**Figure 5:**
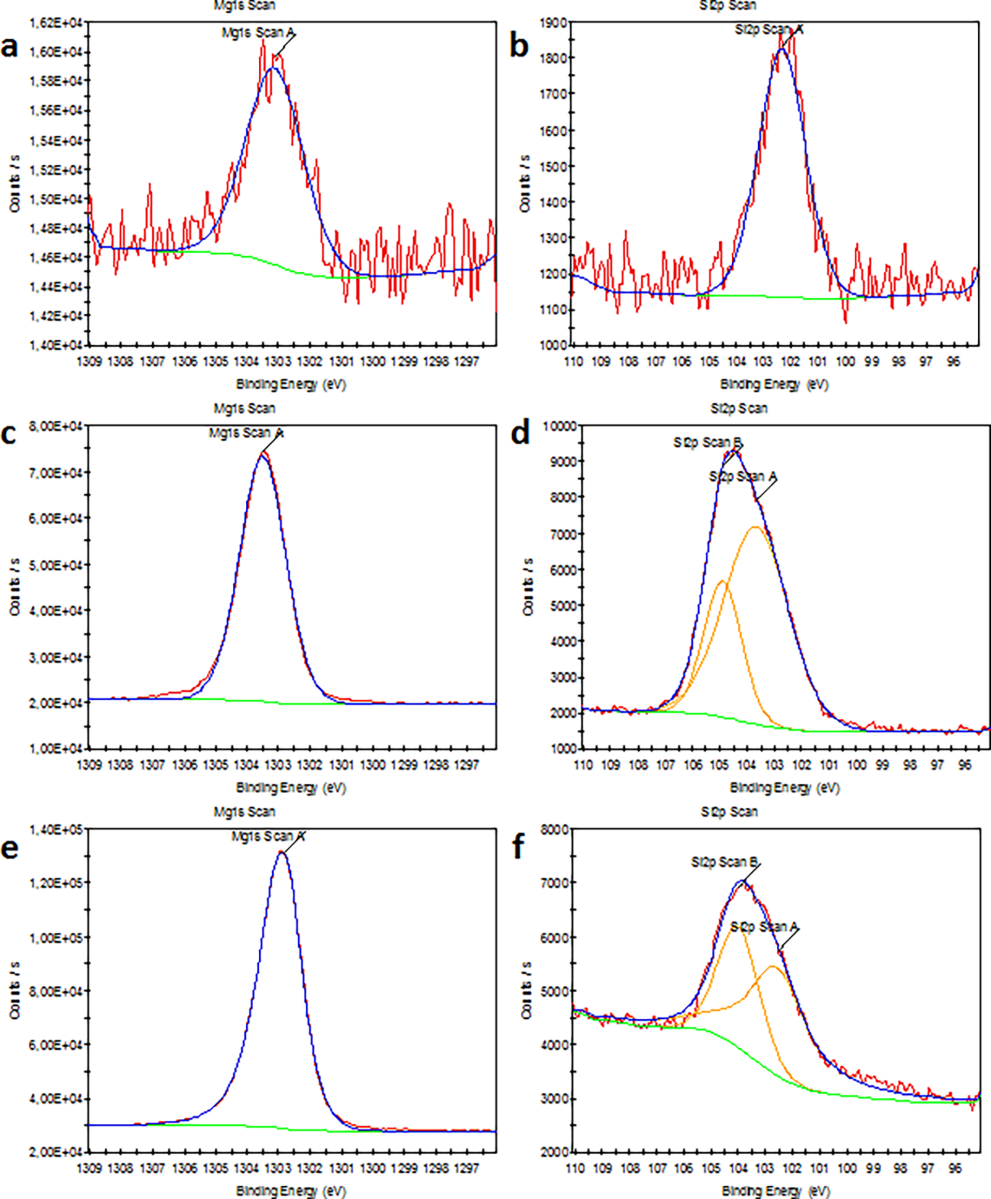
High-resolution XPS spectra of the magnesium and silicon for the structures from (a, b) seed, (c, d) injection, and (e, f) membrane experiments.

**Figure 6:**
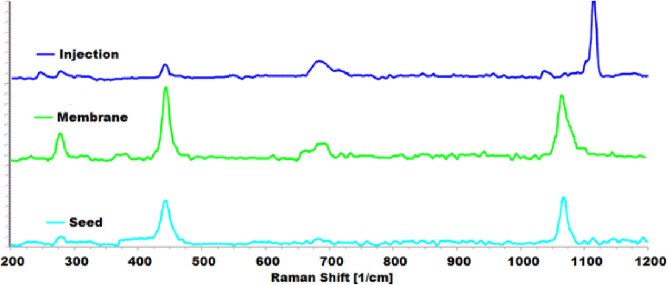
Raman spectra of the structures grown in seed (light blue), injection (dark blue), and membrane (green) experiments.

Another analysis carried out for chemical composition determination was Raman spectroscopy. The Raman spectra of the structures produced in all three growth methods showed three bands at ∼280, ∼440, and ∼690 cm^–1^ (Figure 6). The bands located at 280 and 440 cm^–1^ can be assigned to Mg (OH)_2_. In addition, the band at 690 cm^–1^ was attributed to the Si–O chemical bond.^4,32^ It was clear that the intensities of these bands showed notable differences between structures, which were due to the different orientations and compositions of the samples. This result indicated that the structure produced in the membrane experiment had more Mg and O content, which was consistent with the EDS analysis. The other main peaks observed in all structures were located at 1115, 1065, and 1068 cm^–1^ for injection, membrane, and seed experiments, respectively. These peaks were assigned to Si–O–Si vibrations or C–O stretching motions. C–O band was attributed to the presence of CO_3_^2–^, which was formed due to the carbon dioxide from the air dissolving into solutions.^33,34^ The shift from ∼1070 to ∼1110 cm^–1^ might be a result of conformational variations or the hydroxylation/dehydroxylation process.^35−37^

According to the obtained results, the seed method led to the formation of the most irregular and unstable structures, while membrane growth produced the most standard precipitates in a particular shape due to the dialysis membrane acting as a template. In order to confirm the reproducibility and repeatability of the methods, the experiments were performed at least three times. Seed and injection experiments yielded poor experimental reproducibility due to the unpredictable nature of chemical gardens, random precipitation pattern, and unknown degree of crystallinity. Among these methods, the injection approach demonstrates a higher potential for enhancing controllability by adjusting growth conditions such as solution concentrations, injection rates, and growth directions.^38^ On the other hand, the membrane method yielded more consistent and reproducible standard morphologies compared to the seed and injection experiments. The advantage of this procedure lies in its ability to facilitate growth within a confined area, which decreases the impact of buoyancy and osmosis and prevents the dispersion of precipitates in a disorderly manner. Considering micromorphologies, the microstructures from seed and injection experiments shared significant similarities, as expected. An interesting result about the membrane experiment was that the surface morphologies were clearly different for the two sides of the structure: rough on the solution side, which was rich in Mg and O, and smooth on the side adjacent to the cellulose membrane, which contained a high amount of NaCl. Considering chemical composition, there were no significant differences between the structures produced in different methods. These results indicated that seed and injection techniques provide the production of tubular chemical gardens similar to their examples in nature, whereas the membrane growth method is quite different, enabling the formation of more regular and standard two-dimensional structures. It can provide the production of a wide range of precipitate structures with desired dimensions and surface properties.

## Conclusions

Research on chemical gardens dates back many years, but the scientific interest in these structures has been accelerated for the last few years after they were identified as chemobrionics. The main step in the development of the chemobrionics concept is performing laboratory-scale experiments and providing successful implementation of the obtained results in technological and biological applications. In this article, it was aimed to present a comparative investigation of the different production techniques of chemical gardens at laboratory scale. Seed and injection techniques, which are two common methods in the literature, were performed to obtain three-dimensional chemical garden structures and to show their differences in terms of growth pattern, microstructure, morphology, and chemical composition. In addition, the membrane growth method, which has a very small number of applications for chemical garden production, was employed to highlight how it differs from the other two methods and to add new insights to the existing limited data. The macroscopic observations clearly indicated that all three methods showed different growth patterns, which the membrane method provided the production of most stable and standard structures. Therefore, the application of membrane technique for chemical garden growth could be so suitable for use in material science and electrochemistry research. The micromorphologies did not show any difference between seed and injection methods, but membrane growth generated structures in varying morphologies. The chemical compositions of the chemical gardens were highly similar and mainly included Mg, Si, O, Na, and Cl. Overall, seed and injection techniques have strong potential to be used in research on the emergence of life on other planets since they produce chemical gardens similar to examples in nature, especially hydrothermal chimneys. Also, these methods can be evaluated for the production of chemical gardens to be used in microfluidics, filtration systems, and material science. In order to expand the use of these methods in different fields, they should provide the production of structures with desired characteristics through the implementation of different control strategies. At this point, the membrane growth technique can be more preferable due to its standardization and simplicity. It can provide the production of a wide range of precipitate structures with desired dimensions and surface properties by adjusting the proper membrane and solutions, which should be focused on for further research.

## Supporting Information Available

EDS spectra of structures obtained in the seed experiment, injection experiment, and membrane experiment; full XPS spectra of the structures produced in seed, injection, and membrane experiments; and high-resolution XPS spectra of the oxygen for the structures from seed, injection, and membrane experiments (PDF)
