# Supplementary material for: Comparative Evaluation of Chemical Garden Growth Techniques
Source: Langmuir. 2023 Sep 15;39(38):13611–9. doi: 10.1021/acs.langmuir.3c01681 (PMC10537426; doi:10.1021/acs.langmuir.3c01681)
Supplement: Supplementary file 1 — la3c01681_si_001.pdf [file la3c01681_si_001.pdf]

# Comparative Evaluation of Chemical Garden Growth Techniques

*Bahar ASLANBAY GULER*, Zeliha DEMIREL, Esra IMAMOGLU*

Department of Bioengineering, Faculty of Engineering, Ege University, 35100, Izmir, Turkey

* Corresponding Author: Bahar Aslanbay Guler, baharaslanbay@gmail.com, 91160000820@ogrenci.ege.edu.tr

## Supplementary Information

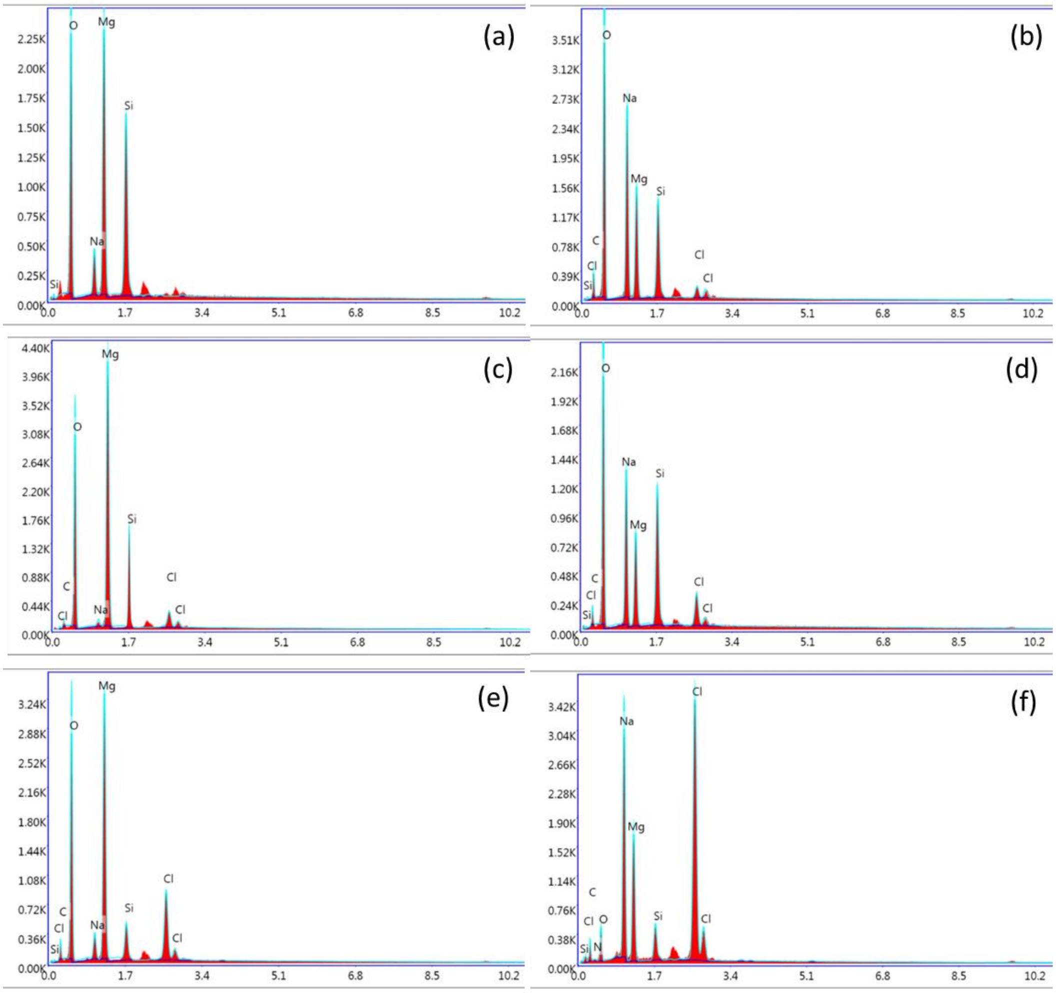


**Figure S1.** EDS spectra of structures obtained in seed experiment (a-external surface, b-interior surface), injection experiment (c-external surface, d-interior surface), and membrane experiment (e- magnesium salt solution side, f- cellulose acetate membrane side).

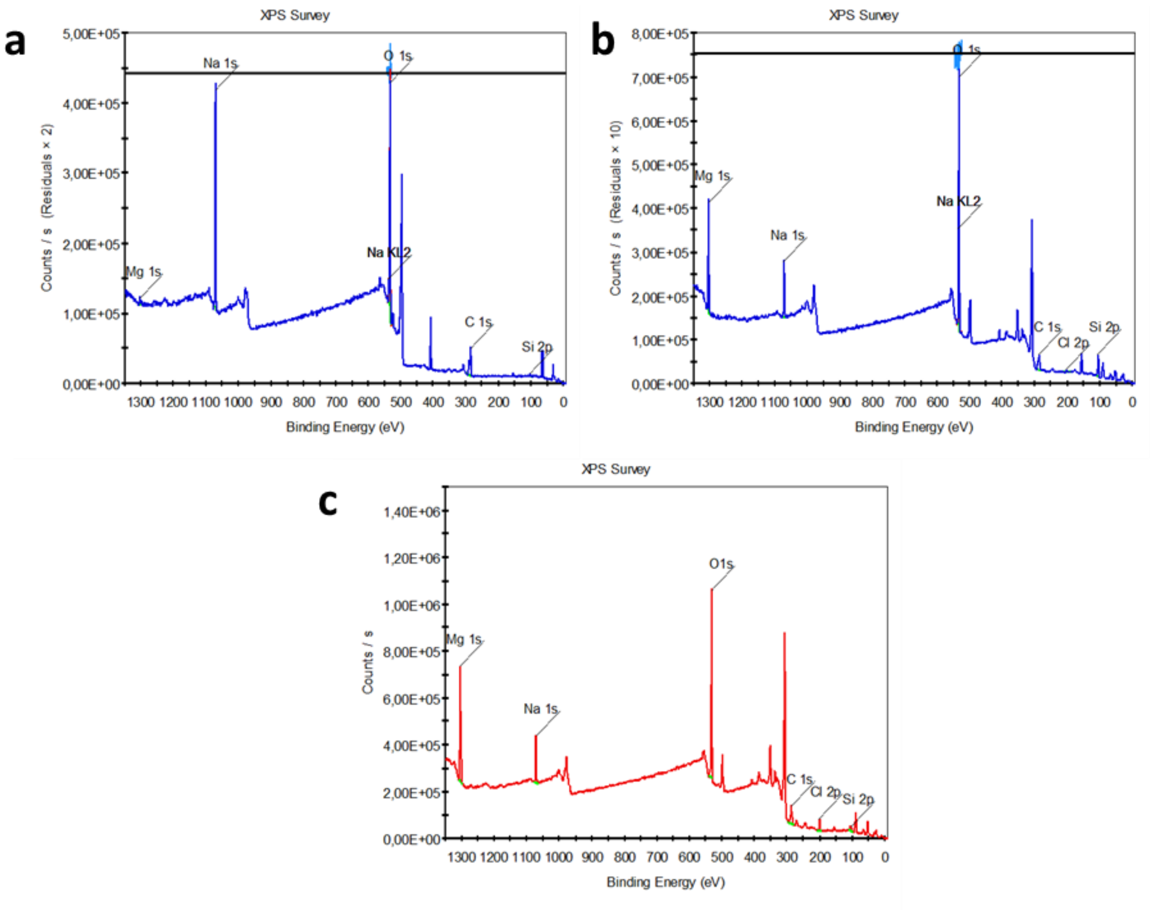


**Figure S2.** Full XPS spectra of the structures produced in a. seed, b. injection, c. membrane experiments

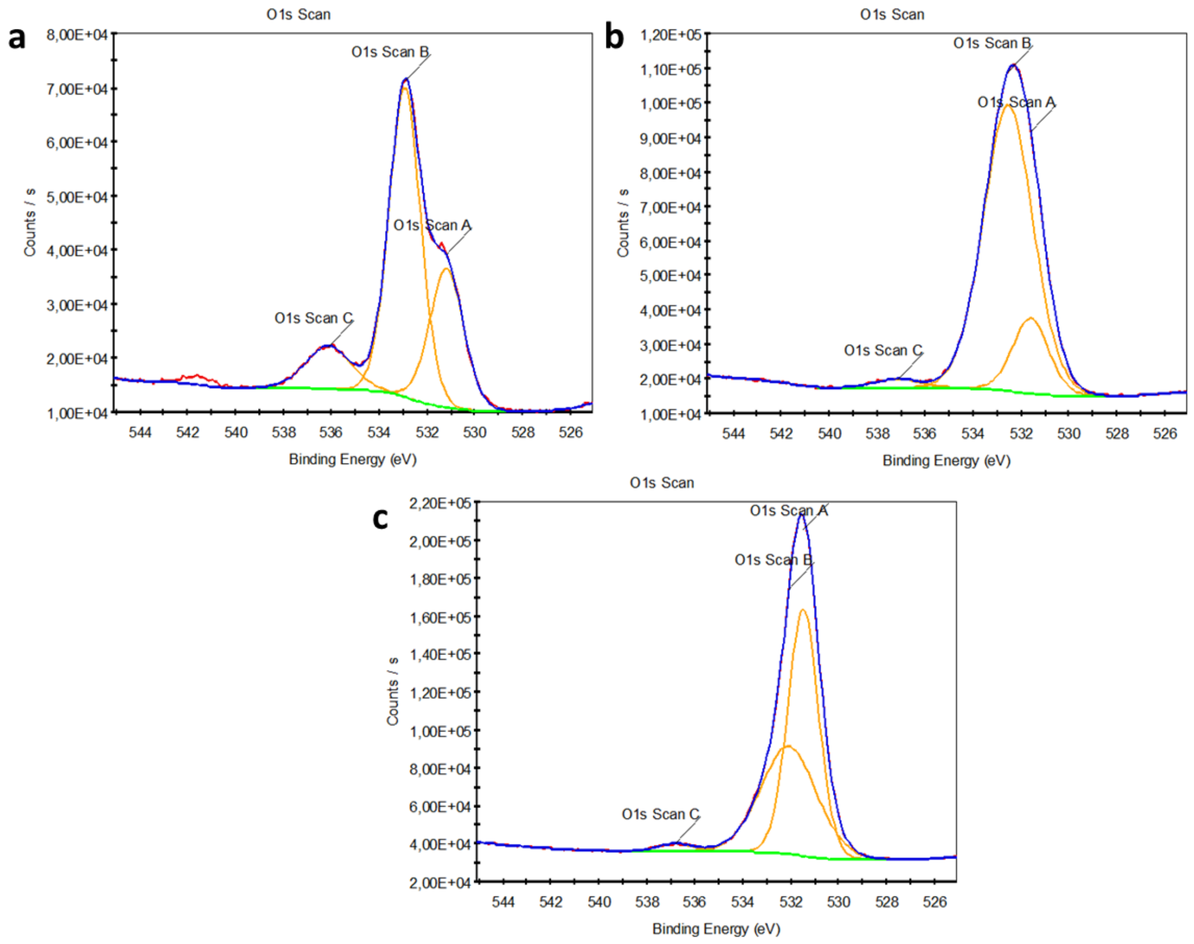


**Figure S3.** High-resolution XPS spectra of the oxygen for the structures from a, seed, b. injection, and c, membrane experiments.
